# Short-Course Rather Than Low-Dose Amphotericin B May Exert Potential Influence on Mortality in Cryptococcal Meningitis Patients Treated With Amphotericin B Plus Flucytosine Alone or in Combination With Fluconazole

**DOI:** 10.3389/fmicb.2019.02082

**Published:** 2019-09-10

**Authors:** Lijun Xu, Ran Tao, Jingjing Wu, Xiahong Dai, Caiqin Hu, Ying Huang, YaoKai Chen, Biao Zhu, Jianqin He

**Affiliations:** ^1^National Clinical Research Center for Infectious Diseases, The First Affiliated Hospital, College of Medicine, Zhejiang University, Hangzhou, China; ^2^State Key Laboratory for Diagnosis and Treatment of Infectious Diseases, The First Affiliated Hospital, College of Medicine, Zhejiang University, Hangzhou, China; ^3^Department of Infectious Diseases, Shulan Hospital, Hangzhou, China; ^4^Chongqing Public Health Medical Center, Chongqing, China

**Keywords:** propensity score matching study, amphotericin, flucytosine, fluconazole, cryptococcal meningitis

## Abstract

**Background:**

The influence of Amphotericin B (AmB) dose and the addition of fluconazole (Flu) on the AmB + 5-flucytosine (5FC) regimen for cryptococcal meningitis (CM) treatment remain debatable.

**Methods:**

A retrospective study was conducted to compare 44 CM patients treated with AmB + 5FC and 78 CM patients treated with AmB + 5FC + Flu using the propensity score matching method. The effects of AmB dosage, AmB course and Flu addition on the cerebrospinal fluid (CSF) chemical profile recovery, adverse effects, and 90-day mortality were compared between the groups.

**Results:**

No differences in adverse effects, the rate of the 14-day CSF chemical profile recovery and 90-day cumulative survival rate (91.2% vs. 87.5%, *P* = 0.637) were observed between the AmB + 5FC group and the AmB + 5FC + Flu group. However, the incidence rates of hypokalemia (33.9%) and creatinine elevation (7.1%) in patients treated with an AmB dosage of 0.4–0.5 mg/kg/d were less than those (53.0 and 22.7%, respectively) treated with an AmB dosage of 0.6–0.7 mg/kg/d (*P* = 0.034 and *P* = 0.018, respectively). The 90-day cumulative survival rate was 70.1% for patients treated with AmB for <14 days and 96.4% for patients treated with AmB for ≥14 days (log-rank *P* < 0.001). Multivariate Cox proportional hazards models suggested the hazard ratio was 26.8 (95% CI: 3.9–183.2) for patients treated with AmB < 14 days than those treated with AmB ≥ 14 days (*P* = 0.001).

**Conclusion:**

Treatment with AmB less than 14 days was associated with a higher 90-day mortality in CM patients. A relative lower dosage but prolonged course of AmB in the +5FC ± Flu regimen led to favorable trends of fewer adverse effects and comparable clinical efficacy.

## Introduction

The current treatment using antifungal drugs against cryptococcosis involves three categories of drugs, namely, amphotericin B (AmB), triazoles, and 5-flucytosine (5FC). The standard protocol for the treatment of cryptococcal meningitis (CM) is divided into three phases: AmB (0.7–1 mg/kg/d) + 5FC (100 mg/kg/d) as an initial 2- to 4-week induction therapy, followed by fluconazole (Flu) 400–800 mg/d for 8 weeks as consolidation therapy and fluconazole 200 mg/d for at least 6 months as subsequent maintenance therapy (Perfect et al., 2010; Perfect and Bicanic, 2015).

There are still some variations in this treatment recommendation. For example, the usage of AmB is usually associated with efficient fungal clearance and an improved survival rate in HIV-infected CM patients (Brouwer et al., 2004). However, many severe side effects such as renal damage, hypokalemia and leukopenia can occur during treatment (Deray, 2002; Bicanic et al., 2015). Furthermore, AmB at times is not available in developing countries (Loyse et al., 2013). Consequently, a short course or low dose of AmB (0.5–0.7 mg/kg/d) is advocated to treat CM patients to increase the tolerance to and decrease the toxicity of AmB (Yao et al., 2005; Fang et al., 2015). However, the effects of shortening the duration or reducing the dose of the induction treatment with AmB in CM patients remains controversial.

Another issue is that the concentration of AmB in the central nervous system is less than 1% of its concentration in serum (Vogelsinger et al., 2006; Kagan et al., 2011; Strenger et al., 2014), whereas the Flu concentration in cerebrospinal fluid (CSF) is up to 60% of the serum concentration (Foulds et al., 1988). Selecting antifungal drugs with high efficacy and/or penetration can improve clinical outcomes of fungal infections, including *Cryptococcus* infection (Kethireddy and Andes, 2007; Felton et al., 2014; Benhamou et al., 2018). For example, some doctors prefer a regimen of AmB + 5FC + Flu as the induction therapy (Xu et al., 2018). However, whether adding Flu to the AmB + 5FC regimen can improve the survival rate of patients remains under debate.

We conducted a retrospective study to observe the effects of the AmB course (<14 days vs. ≥14 days), AmB dose (0.4–0.5 mg/kg/d vs. 0.6–0.7 mg/kg/d), and Flu supplementation on the clinical outcomes of CM patients treated with AmB + 5FC to delineate the optimal antifungal regimen for CM patients.

## Materials and Methods

### Diagnostic Criteria

The diagnosis of CM was defined as the presence of at least one of the following criteria: (1) culture of cerebrospinal fluid (CSF) positive for *Cryptococcus neoformans*, (2) CSF India ink smear of centrifuged sediment positive for *Cryptococcus*, (3) histopathology compatible with *Cryptococcus* (5- to 10-μm encapsulated yeasts observed in brain tissue using silver and/or PAS stain), or (4) probable CM with the clinical syndrome of meningitis and positive cryptococcal antigen titer ≥1:4 or positive CrAg lateral flow assay in CSF even without microbiological or pathological documentation.

The *Cryptococcus* counting procedure is explained briefly as follows: 1 ml of CSF was collected and centrifuged at a speed of 3000 rpm × 10–15 min. Then, the sediment (≈100 μl) was mixed with a small drop of India ink. A large coverslip was applied over 100 μl of mixture on the glass slide and pressed gently to obtain a thin mount. The slide was scanned under a low power field in reduced light using a microscope, then switched to a high power field (HPF) for counting if cryptococcal capsules were found. The *Cryptococcus* count (cells/HPF) = (total number of *Cryptococcus* capsules in 10 HPFs)/10.

Hypokalemia was defined as serum potassium less than 3.0 mmol/L. Anemia was defined as a concentration of hemoglobin less than 110.0 g/L, and creatinine elevation was defined as a serum creatinine level higher than 110.0 mmol/L.

### Study Cohort and Patient Enrollment

**FIGURE 1:**
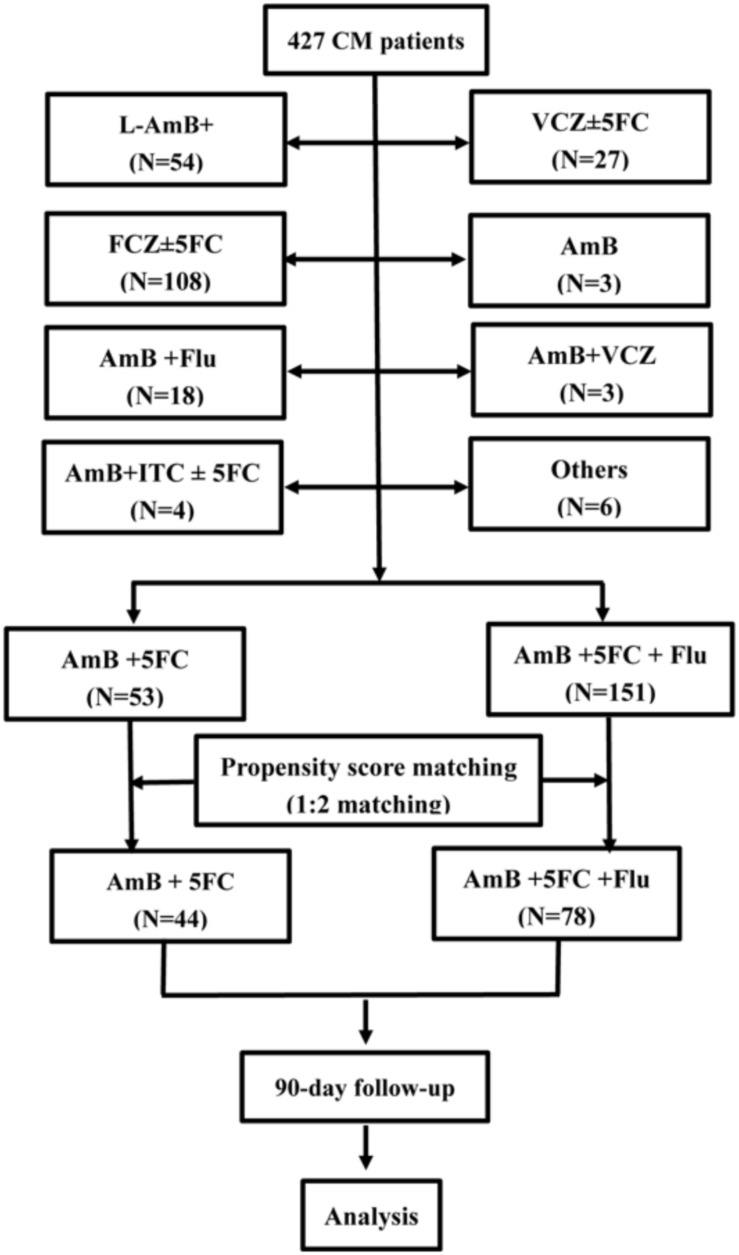
Flowchart of patient selection.

A total of 427 CM patients treated at the First Affiliated Hospital, College of Medicine, Zhejiang University between January 2007 and December 2017 were eligible for this retrospective study. Of those, 124 (26.1%) had human immunodeficiency virus (HIV) infection, 81 (17.0%) had hepatitis B virus (HBV) infection, 41 (8.6%) had liver cirrhosis, 52 (10.9%) had diabetes, 19 (4.0%) had chronic kidney disease (CKD), 14 (2.9%) had chronic hematological disease (CHD), 10 (2.1%) had malignancies, 28 (5.9%) underwent solid organ transplantation (SOT), and 37 (7.8%) had autoimmune diseases. Overall, HIV-uninfected CM patients were predominant (73.9%). There were 27 patients treated with voriconazole (VCZ) ± 5FC as the induction therapy, 3 with mono-AmB, 3 with AmB + VCZ, 53 with AmB + 5FC, 54 with a liposomal amphotericin B (L-AmB)-based regimen, 108 with Flu ± 5FC, 151 with AmB + 5FC + Flu, 18 with AmB + Flu, 4 with AmB + Itraconazole (ITC) ± 5FC, and 6 with other regimens. Fifty-three patients treated with AmB + 5FC and 151 patients treated with AmB + 5FC + Flu were eligible for observation after the exclusion of patients treated with other antifungal regimens. Propensity score matching (PSM) for age, sex, body mass index (BMI), predisposing diseases, routine blood test results, liver function, and renal function was used to select patients according to a match ratio of 1:2. There were 44 patients treated with the induction treatment of AmB + 5FC, and 88 patients (including 10 repeated cases) treated with AmB + 5FC + Flu were selected for study. Finally, 44 patients who received induction treatment with AmB + 5FC and 78 patients who received initial treatment with AmB + 5FC + Flu were enrolled in the present study after exclusion of 10 repeated cases. The enrollment was performed according to the process indicated in Figure 1.

### Antifungal Treatment in Induction Therapy

Forty-four patients were initially treated with amphotericin B deoxycholate (AmB) + 5FC, and 78 were initially treated with AmB + 5FC + Flu. According to the patients’ tolerance, AmB that was administered to patients ranged from 30 to 60 mg/d (approximately 0.4–0.7 mg/kg/d) via peripheral intravenous (IV) in 5% dextrose in a total volume of 500 ml. One liter of saline was administered intravenously immediately prior to amphotericin administration to minimize renal toxicity. The oral 5FC dosage was 80–100 mg/kg/d, and the Flu dosage was from 400 to 800 mg/d via IV. The mean dose of AmB was 0.4–0.5 mg/kg in 56 (45.9%) patients and 0.6–0.7 mg/kg in 66 (54.1%) patients whose dosage of AmB was 0.4–0.7 mg/kg/d during induction treatment. There were 42 patents treated with AmB for less than 14 days. Of those, 4 patients died, and 38 patients had adverse effects. The AmB treatment duration was 6.0 (3.0–13.0) days for patients treated with AmB for less than 14 days and 29.5 (19.0–39.8) days for patients treated with AmB for more than 14 days.

### Follow-Up and Collection of Clinical Data

Basic medical information of the patients (such as name, sex, BMI, blood test, CSF profiles, imaging examination, and treatments, etc) was recorded and saved in the electronic medical records system (EMRS) of the hospital. The time to follow-up was calculated from the first day of antifungal treatment. Patients were followed up for 90 days.

### Statistical Analyses

Continuous normal variables are presented as the means ± standard deviations, and continuous abnormal variables are presented as the medians (interquartile ranges, IQRs). Categorical variables are presented as the number of cases (percentage). Continuous variables were compared using the Student’s *t*-test or Mann-Whitney *U* test. Categorical variables were compared using the χ2 analysis or Fisher’s exact test. Patient survival was analyzed using the Kaplan–Meier method. CM-related death was defined as an “event.” Data for patients were censored at the date of the final visit (for those alive at the end of the follow-up period), the date last known to be alive (for those with unknown vital signs) or the date of death from a cause that was not known to be AIDS related. Patients were followed up until 10 weeks after initiation of therapy. These covariates were first analyzed using the univariate Cox model. Thereafter, covariates with *P* < 0.2 in the univariate model were selected for the multivariate Cox proportional hazards model using the forward stepwise (likelihood ratio) method. The missing data were excluded from the multivariate analysis. Covariates (sex, age, BMI, white blood cell, hemoglobin, platelets, alanine aminotransferase, aspartate aminotransferase, serum creatinine, serum urea, CSF profile, AmB dose, and course) were used for propensity matching with a matching tolerance of 0.1 according to the match ratio of 1:2. *P* < 0.05 (two-tailed) was considered significant. Data analyses were performed using SPSS version 24.0 (IBM, Armonk, NY, United States) and GraphPad Prism version 6.0 (GraphPad Software, La Jolla, California, United States).

### Ethical Approval of the Study Protocol

The study protocol was in accordance with the Declaration of Helsinki 1975 guidelines and was approved by the Ethics Committee of the First Affiliated Hospital, School of Medicine, Zhejiang University (Hangzhou, China) (No. 2017-688). Written informed consent was obtained from all the patients, and all data analyzed were anonymous.

## Results

### Comparison of Demographic, Clinical, and Laboratory Characteristics

Cryptococcal meningitis was diagnosed in 55 individuals via the method of CSF India ink staining and culture after admission, 20 by sole CSF *Cryptococcus* culture positivity, 4 by CSF *Cryptococcus* culture positivity combined with either cryptococcal antigen titer ≥1:4 or CrAg lateral flow assay in CSF, 24 by India ink staining combined with either cryptococcal antigen titer ≥1:4 or CrAg lateral flow assay in CSF, and 19 patients by cryptococcal antigen titer ≥1:4 or positive CrAg lateral flow assay in CSF but without the finding of *Cryptococcus*.

Forty-four of the 53 patients who received induction treatment with AmB + 5FC and 78 of the 151 who received induction treatment with AmB + 5FC + Flu were enrolled after PSM. Of those patients, 19 (43.2%) were treated with AmB for less than 14 days, and 25 (56.8%) were treated with AmB for ≥14 days in the AmB + 5FC group; 23 (29.5%) were treated with AmB for <14 days, and 55 (70.5%) were treated with AmB for ≥14 days in the AmB + 5FC + Flu group (*P* = 0.126). Similarly, 36.4% of the patients were treated with AmB doses ≤ 0.5 mg/kg/d, and 63.6% were treated with AmB doses > 0.5 mg/kg/d in the AmB + 5FC group; 51.3% of the patients were treated with AmB doses ≤ 0.5 mg/kg/d, and 48.7% were treated with AmB doses > 0.5 mg/kg/d in the AmB + 5FC + Flu group (*P* = 0.112). The CSF cryptococcal fungal burden was 2 (0–5) cells/HPF in patients treated with AmB + 5FC group and 2 (0–4) cells/HPF in patients treated with AmB + 5FC + Flu (*P* = 0.497). There were 15 HIV-infected (12.3%) and 107 HIV-uninfected CM patients (87.7%) in our cohort. The demographic characteristics and laboratory test results are listed in Table 1.

**TABLE 1:** Comparisons of the clinical features of CM patients treated with AmB + 5FC and those treated with AmB + 5FC + Flu.

**Factors**	**Patients treated with AmB + 5FC**		***P* value**
	**Without Flu (*N* = 44)**	**With Flu (*N* = 78)**	
Sex (male %)	25 (56.8)	50 (64.1)	0.427
Age (years)	50.7 ± 14.1	47.8 ± 14.7	0.291
BMI	21.6 ± 3.2	20.9 ± 4.5	0.450
**Predisposing conditions**			
HBV	6 (13.6)	13 (20.5)	0.658
HIV	6 (13.6)	9 (11.5)	0.735
Liver cirrhosis	3 (6.8)	4 (5.1)	0.702
Diabetes	4 (9.1)	6 (7.7)	0.746
CKD	1 (2.3)	2 (2.6)	1.000
CHD	2 (4.3)	3 (3.8)	1.000
SOT	1 (2.3)	0	0.361
Autoimmune disease	2 (4.5)	9 (11.5)	0.324
Malignancies	1 (2.3)	4 (5.1)	0.653
Steroid usage	5 (11.4)	11 (14.1)	0.667
Cytotoxic drug usage	2 (4.3)	2 (2.6)	0.621
**Routine blood test**			
WBC (×10^9^/L)	7.0 (5.1–9.6)	7.7 (4.8–9.7)	0.668
Hemoglobin (g/L)	124.9 ± 27.0	125.3 ± 22.6	0.937
Platelets (×10^9^/L)	201.6 ± 79.5	206.7 ± 66.4	0.706
**Liver/renal function**			
Serum albumin (g/L)	40.9 ± 5.7	40.5 ± 6.4	0.750
ALT (U/L)	19.0 (11.0–32.0)	21 (14.0–33.0)	0.233
AST (U/L)	15.0 (12.3–22.0)	16.0 (13.0–24.0)	0.338
Creatinine (μmol/L)	58.5 (47.3–67.0)	57.5 (45.0–70.3)	0.915
Urea (mmol/L)	4.5 (3.2–5.3)	4.1 (3.2–5.9)	0.658
C-reactive protein	3.3 (1.8–12.4)	4.9 (2.4–15.1)	0.262
**CSF profile**			
*Cryptococcus* count (/HPF)	2 (0–5)	2 (0–4)	0.497
Culture positive rate (%)	75.0	64.3	0.164
Glucose (mmol/L)	2.1 ± 1.2	2.2 ± 1.4	0.508
Protein (g/L)	0.8 (0.6–1.2)	0.8 (0.5–1.4)	0.543
Chlorine (mmol/L)	115.1 ± 7.6	116.5 ± 6.4	0.300
WBC (×10^6^/L)	85 (18–160)	50 (11–125)	0.208
Intracranial pressure (mmH_2_O)	250 (196–377)	250 (169–350)	0.215
**Treatment**			
AmB < 14 days (%)^#^	19 (43.2)	23 (29.5)	0.126
AmB ≤ 0.5 mg/kg/d (%)^$^	20 (36.4)	40 (51.3)	0.112

*ALT, alanine aminotransferase; AmB, amphotericin B; AST, aspartate aminotransferase; BMI, body mass index; CKD, chronic kidney disease; CHD, chronic hematological disease; CSF, cerebrospinal fluid; HBV, hepatitis B virus; HIV, human immunodeficiency virus; HPF, high power field; SOT, solid organ transplantation; WBC, white blood cells. **#**, the median dosage was 0.5 mg/kg/d; $, the median course was 16.5 days.*

### Adverse Effects of Treatments

The main adverse effects that were observed in our study were hypokalemia, anemia, renal damage, and chills. Of the patients, 54 (44.3%) presented with hypokalemia, 34 (27.9%) patients had anemia, 19 (15.6%) had creatinine elevation, and 3 (2.5%) patients had chills during treatment. No differences were found in the incidence of hypokalemia (43.2% vs. 44.9%, *P* = 0.857), anemia (27.3% vs. 28.2%, *P* = 0.912), creatinine elevation (22.7% vs. 11.5%, *P* = 0.102), or chills (4.5% vs. 1.3%, *P* = 0.295) between the AmB + 5FC group and the AmB + 5FC + Flu group, respectively.

The overall incidence of hypokalemia was 33.9% (19/56) in patients treated with AmB doses of 0.4–0.5 mg/kg/d and 53.0% (35/66) in those treated with AmB doses of 0.6–0.7 mg/kg/d (*P* = 0.034). The overall incidence of creatinine elevation was 7.1% (4/56) in patients treated with AmB doses 0.4–0.5 mg/kg/d and 22.7% (15/66) in patients treated with AmB doses of 0.6–0.7 mg/kg/d (*P* = 0.018). However, the incidence of anemia was 23.2% (13/56) in patients treated with AmB doses of 0.4–0.5 mg/kg/d and 36.4% (21/66) in patients treated with AmB doses of 0.6–0.7 mg/kg/d (*P* = 0.291).

There were 42 patents treated with AmB for less than 14 days, namely, 19 patients in the AmB + 5FC group and 23 patients in the AmB + 5FC + Flu group (*P* = 0.126). In the AmB + 5FC group, 17 patients discontinued AmB treatment because of adverse effects, and 2 patient discontinued AmB treatment because of death. Furthermore, in the AmB + 5FC + Flu group, 21 patients discontinued AmB treatment because of adverse effects, and 2 patients discontinued AmB treatment because of death. Among the patients who survived more than 14 days, the percentage who received AmB for <14 days was 40.5% (17/42) in the AmB + 5FC group and 27.6% (21/76) in the AmB + 5FC + Flu group (*P* = 0.153).

### Two-Week CSF Profile Change

The CSF protein level in the AmB + 5FC group was 0.8 (0.6–1.2) g/L at admission and decreased to 0.6 (0.5–0.9) g/L after 2 weeks of antifungal treatment. In addition, the CSF protein level in the AmB + 5FC + Flu group was 0.8 (0.5–1.4) g/L at admission and decreased to 0.6 (0.4–0.9) g/L after 2 weeks of antifungal treatment. There were no significant differences in the protein level reduction between the two groups [AmB + 5FC group: −0.2 (−0.4–0) g/L vs. AmB + 5FC + Flu group: −0.2 (−0.6–0) g/L; *P* = 0.804]. Similarly, no differences in glucose and chlorine level increase were observed between the AmB + 5FC and AmB + 5FC + Flu groups [Glucose (mmol/L): 0.7 ± 1.3 vs. 0.6 ± 1.1, respectively, *P* = 0.684; chlorine (mmol/L): 4.4 ± 5.7 vs. 4.2 ± 7.0, respectively, *P* = 0.866).

In addition, the 2-week CSF *Cryptococcus* culture sterilization was 69.2% (18/26) in the AmB + 5FC group and 75.9% (22/29) in the AmB + 5FC + Flu group (*P* = 0.581) among those patients with data available.

### Anti-fungal Treatment and 90-Day Survival Rate

**FIGURE 2:**
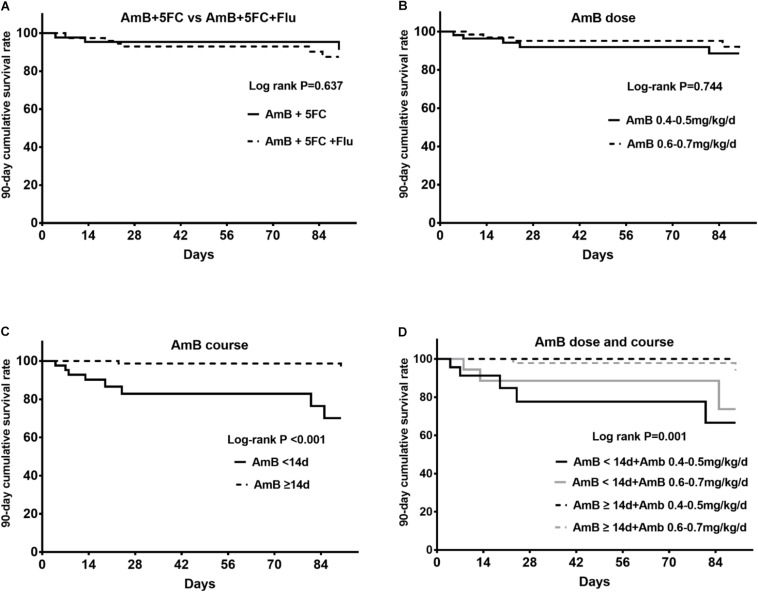
**(A)** The 90-day cumulative survival rates between the AmB + 5FC group and the AmB + 5FC + Flu group were similar (*P* = 0.637). **(B)** The 90-day cumulative survival rate in patients treated with AmB doses of 0.3–0.5 mg/kg/d and 0.5–0.7 mg/kg/d (*P* = 0.744). **(C)** The 90-day cumulative survival rate in patients treated with AmB for <14 days and ≥14 days (log-rank *P* < 0.001). **(D)** Combined effects of the AmB dose and course on the 90-day cumulative survival rate of patients (log-rank *P* = 0.001).

Ten patients (8.2%) died within 90 days; 4 patients died within 14 days, and 6 patients died in 15–90 days after antifungal treatment. Of those patients, there were 3 patients in the AmB + 5FC group and 7 patients in the AmB + 5FC + Flu group. The 90-day cumulative survival rate was 91.2% in patients treated with AmB + 5FC and 87.5% in patients treated with AmB + 5FC + Flu (*P* = 0.637) (Figure 2A). We further analyzed the effect of the AmB dose on patients’ survival rates. Patients were categorized into two groups according to whether they received AmB doses of 0.4–0.5 mg/kg/d or 0.6–0.7 mg/kg/d. Overall, we found that the 90-day cumulative survival rate was 88.7% for patients treated with an AmB dose of 0.4–0.5 mg/kg/d and 89.1% for patients treated with an AmB dose of 0.6–0.7 mg/kg/d (log-rank *P* = 0.744) (Figure 2B).

Next, we analyzed the effect of the AmB treatment course on patients’ survival rates. Our data suggested that the cumulative survival rate was 70.1% for patients treated with AmB for less than 14 days and 96.4% for patients treated with AmB for ≥14 days among our cohort (log-rank *P* < 0.001) (Figure 2C).

Furthermore, the AmB dose (0.4–0.5 mg/kg/d vs. 0.6–0.7 mg/kg/d) and AmB course (<14 days *vs*. ≥14 days) were taken together for analysis. We found that the 90-day cumulative rate was 100% for patients treated with an AmB course ≥14 days along with an AmB dose of 0.4–0.5 mg/kg/d, 93.9% for patients treated with an AmB course ≥14 days along with an AmB dose of 0.6–0.7 mg/kg/d, 73.8% for patients treated with an AmB course <14 days along with an AmB dose of 0.6–0.7 mg/kg/d and 66.6% for patients treated with an AmB course <14 days along with an AmB dose of 0.4–0.5 mg/kg/d (log-rank *P* = 0.001) (Figure 2D).

### Risk Factors Associated With 90-Day Mortality

We analyzed the factors associated with death in CM patients. We stratified patients according to the following criteria: sex (male and female), age (years; <50.0 and ≥50.0), C-reactive protein level (CRP) (mg/L; <10.0 and ≥10.0), WBC count (×10^9^/L; <10.0 and ≥10.0), hemoglobin (g/L; <110.0 and ≥110.0), serum albumin (g/L; <35.0 and ≥35.0), intracranial pressure (ICP) (mmH_2_O; <250.0 and ≥250.0), CSF WBC count (×10^6^/L, <30.0 and ≥30.0), CSF glucose (mmol/L; <1.5 and ≥1.5), CSF chlorine (mmol/L; <105.0 and ≥105.0), CSF total protein (g/L; <0.9 and ≥0.9), CSF fungal count (cells/HPF; <20.0 and ≥20.0), induction treatment (AmB + 5FC vs. AmB + 5FC + Flu), AmB dose (mg/kg/d; 0.4–0.5 and 0.6–0.7), and AmB course (days; <14.0 and ≥14.0). In the unadjusted univariate model, our data suggested that a WBC count in peripheral blood ≥10 × 10^9^/L (*P* = 0.050), CSF chlorine < 105.0 mmol/L (*P* = 0.005), CSF fungal count ≥20.0 capsules/HPF (*P* = 0.044), and an AmB course <14 days (*P* = 0.002) were associated with 90-day mortality after the diagnosis of CM. CSF WBC < 30.0 × 10^6^/L (*P* = 0.161), CSF glucose < 1.5 mmol/L (*P* = 0.113), and CSF protein (*P* = 0.084) were close to 90-day mortality. However, sex, age, CRP, hemoglobin, serum albumin, ICP, AmB + 5 FC ± Flu, and AmB dose were not associated with an increase in CM-related mortality. In the multivariate Cox proportional hazard model, only the WBC count in peripheral blood, CSF chlorine < 105.0 mmol/L and an AmB course <14.0 days in anti-fungal treatment were still associated with the 90-day cumulative survival rate. The hazard ratios (HRs) were 25.1 (95% CI: 3.7–171.1) for patients with CSF chlorine < 105.0 mmol/L compared with that of patients with CSF chlorine ≥105.0 mmol/L (*P* = 0.001), 5.0 (1.1–23.3) for patients with a WBC count in peripheral blood ≥10.0 × 10^9^/L compared with that of patients with a WBC count in peripheral blood <10.0 × 10^9^/L (*P* = 0.042) and 24.4 (95% CI: 3.6–163.6) for patients treated with AmB < 14.0 days compared with those treated with AmB ≥ 14.0 days (*P* = 0.001) (Table 2).

**TABLE 2:** Risk factors for 10-week mortality in CM patients in univariate/multivariate Cox proportional hazards models.

**Factor**	**Number (*n* = 122)**	**CM deaths (*n* = 9)**	**Univariate**		**Multivariate**	
			**HR (95% CI)**	***P* value**	**HR (95% CI)**	***P* value**
**Sex**						
Male	75	7(9.3)	0.5 (0.2–2.6)	0.547		–
Female	47	3(6.4)	1			
**Age (years)**						
<50.0	71	5(7.0)	1	0.487		–
≥50.0	51	5(9.8)	1.6 (0.5–5.5)			
**CRP (mg/L)**						
<10.0	85	6(7.1)	1	0.496		–
≥10.0	37	4(10.8)	1.5 (0.4–5.5)			
**WBC (×10^9^/L)**						
<10.0	101	6(5.9)	1	0.050	1	0.031
≥10.0	21	3(14.3)	3.5 (1.0–12.6)		5.5 (1.2–25.6)	
**Hemoglobin (g/L)**						
<110.0	23	3(13.0)	1.9 (0.5–7.5)	0.304		–
≥110.0	99	7(7.1)	1			
**Albumin (g/L)**						
<35.0	18	1(5.6)	0.7 (0.1–6.0)	0.791		–
≥35.0	104	9(8.7)	1			
**ICP (mmH_2_O)**						
<250.0	63	3(4.8)	1	0.244		–
≥250.0	59	7(11.9)	2.2 (0.6–8.8)			
**CSFWBC (×10^6^/L)**						
Missing data	2	1				0.085
<30.0	51	6(10.9)	2.7 (0.7–10.8)	0.161		
≥30.0	69	3(6.3)	1.0			
**CSFglucose (mmol/L)**						
Missing data	2	0				0.252
<1.5	42	6(11.8)	2.8 (0.8–9.9)	0.113		
≥1.5	78	4(5.1)	1			
**CSF chlorine (mmol/L)**						
Missing data	2	0				
<105	10	3(30.0)	7.2 (1.8–28.3)	0.005	23.1 (3.5–153.2)	0.001
≥105	110	7(6.4)	1		1	
**CSF total protein (g/L)**						
<0.9	67	3(4.7)	1			0.152
≥0.9	55	7(13.0)	3.3 (0.9–12.8)	0.084		
**CSF fungal count (cells/HPF)**						
<20.0	102	6(5.9)	1			0.448
≥20.0	20	4(20.0)	3.7 (1.0–13.1)	0.044		
**Induction treatment**						
AmB + 5FC	44	3(6.8)	1			–
AmB + 5FC + Flu	78	7(9.0)	1.4 (0.4–5.4)	0.639		
**AmB dose (mg/kg/d)**						
0.4–0.5	56	5(8.9)	1.2 (0.4–4.2)	0.744		–
0.6–0.7	66	5(7.6)	1			
**AmB course (days)**						
AmB < 14.0	42	5(11.9)	11.4 (2.4–54.2)	0.002	26.8 (3.9–183.2)	0.001
AmB ≥ 14.0	80	4(5.0)	1		1	

*CM, cryptococcal meningitis; HR, hazard ratio; AmB, amphotericin B; 5FC, flucytosine; Flu, fluconazole.*

## Discussion

Although AmB plus 5FC followed by a switch to Flu is the standard treatment for CM, there are some challenges to its implementation in clinical practice. For example, some patients cannot endure the side effects of AmB, which leads to course shortening or dose reduction in the application of AmB. In addition, the concentration of AmB in the CSF is only 1% of the serum concentration (Vogelsinger et al., 2006; Kagan et al., 2011; Strenger et al., 2014), whereas the fluconazole concentration in the CSF is approximately 60% of the serum concentration (Foulds et al., 1988). Thus, we sought to determine whether AmB + 5FC combined with Flu could improve the clinical outcome of CM patients in clinical practice.

In our present study, we compared the efficacy of AmB plus 5FC alone or supplemented with Flu in Chinese CM patients, and we found the following: (1) adding Flu to the AmB + 5FC regimen did not improve the recovery of the 2-week CSF chemical profile nor did it improve the 90-day survival rate of the patients; (2) treatment with AmB for less than 14 days increased the risk of death for CM patients, especially patients treated with an AmB dosage of 0.4–0.5 mg/kg/d; and (3) an AmB dosage of 0.4–0.5 mg/kg/d in the AmB + 5FC ± Flu regimen led to a lower incidence of adverse effects but could achieve clinical efficacy that was comparable to the results of a dosage of AmB 0.6–0.7 mg/kg/d.

Flu is usually used as consolidation treatment for *Cryptococcus* infection. In some cases, Flu ± 5FC can be used as an alternative induction treatment for patients when AmB is unavailable or unendurable. Particularly, high-dose fluconazole plus 5FC was reported as an alternative treatment for HIV-infected CM patients (Bicanic et al., 2008; Nussbaum et al., 2010). However, high-dose fluconazole monotherapy is insufficient for the treatment of HIV-infected CM patients (Beyene et al., 2017). To date, studies involving Flu as a CM treatment were mainly performed in HIV-infected populations; thus, the effectiveness of Flu in CM patients with other predisposing diseases remained unclear. In our present study, we enrolled 44 patients whose induction treatment was AmB + 5FC and 78 patients whose induction treatment was AmB + 5FC + Flu by a PSM method to balance the basic characteristics of the patients. We found that the combination of AmB + 5FC + Flu as induction therapy did not enhance the recovery of the CSF biochemical profile and did not increase 2-week CSF *Cryptococcus* culture sterilization; furthermore, this regimen did not decrease the 90-day mortality rate of CM patients. A recent published study indicated that triple therapy (AmB + 5FC + Flu) had a higher incidence of satisfactory outcomes and a higher rate of *Cryptococcus* sterilization in the CSF than did therapy with AmB + 5FC (Xu et al., 2018). Our study results coincided with those of previous studies (Yao et al., 2014) and those of a newly published study (Molloy et al., 2018) in that the addition of a standard dose of Flu to the AmB + 5FC regimen did not improve the clinical outcomes of CM patients. One possible reason was that Flu contradicted the anti-*Cryptococcus* activity of AmB in an *in vitro* study (Santos et al., 2012). Another possible reason for the observed discrepancies between our study and Xu’s study may be that first, the predisposing conditions of the CM patients were different. In our study, 12.3% of the patients included were HIV-infected, but HIV-infected patients were excluded in Xu’s study. Other conditions, such as the use of steroids and cytotoxic drugs, were considered in our research. Furthermore, the discrepancies between the predisposing conditions in the CM patients were diminished to some extent by the PSM method used in our study. Second, the number of individuals in these studies (including ours), do not represent large populations, which could hide some associations between antifungal therapy regimens and the patients’ outcomes.

Interestingly, our study indicated that short-term usage of AmB (<14 days) was associated with increased mortality in CM patients. The hazard ratios were 27 times higher for patients treated with AmB for less than 14 days than for those who were treated with AmB for ≥14 days in patients treated with AmB + 5FC ± Flu. Our study results were consistent with previous recommendations that AmB + 5FC should be administered at least for 2 weeks (Perfect et al., 2010). However, it should be clear that the short-term usage of AmB also improved the clinical outcome of CM patients compared with that of patients not treated with AmB (Jackson et al., 2012; Muzoora et al., 2012).

Another interesting finding was that the lower AmB dosage had less impact on the survival of patients than did the course of treatment. Compared to dosages of AmB 0.6–0.7 mg/kg/d, dosages of AmB 0.4–0.5 mg/kg/d were not related to increased mortality in CM patients but were related to a lower incidence of adverse effects such as hypokalemia and creatinine elevation. It was noted that a low dosage of AmB (including liposomal AmB) produced more safety and similar efficacy to prevent or treat fungal infections (Koh et al., 2002; Lequaglie, 2002; Kassamali et al., 2013; Yasu et al., 2018). The possible reason that a low dosage of AmB achieved comparable efficacy to a standard dosage of AmB may be that first, AmB is a fungicidal medicine with a long half-life. The elimination half-life of AmB is approximately 15 days, and excretion time is over weeks to months (Atkinson and Bennett, 1978; Bellmann and Smuszkiewicz, 2017). Thus, a slow-released depot of low-dose AmB in the body probably exceeded the minimum concentration against *Cryptococcus*. Second, higher serum AmB concentration does not necessary relate to higher CSF concentration. The AmB concentration in CSF is only 1% of its concentration in serum (Vogelsinger et al., 2006; Strenger et al., 2014); consequently, variance in the serum AmB concentration had less impact on the CSF AmB concentration. In addition, low CSF levels of AmB did not necessarily predict low brain tissue levels following liposomal AmB infusion. The animal studies demonstrated a 30-fold decrease in AmB concentration from plasma to brain tissue and a further 100-fold decrease from brain tissue to CSF levels after seven daily doses of liposomal AmB (Groll et al., 2000). Third, the passage of AmB from serum into CSF is a slow process. Increasing the AmB course will increase the AmB transfer rate from serum to CSF (Strenger et al., 2014).

Given the tolerance to and accessibility of AmB, a low dosage of AmB (such as 0.5 mg/kg/d) as induction treatment for *Cryptococcus* infection could be considered for CM patients. Notably, the 90-day mortality of 8.2% in our patients is significantly lower than that of approximately 35% in African CM patients (Molloy et al., 2018), 32% in Taiwanese CM patients (Lee et al., 2011), and 15–25% in American CM patients (Liao et al., 2012), indicating that a lower dose but longer course of AmB did not necessarily reduce the survival rate of CM patients. However, it also should be noted that the majority of the CM population were HIV-uninfected rather than HIV-infected, and a study from China indicated that HIV-uninfected CM patients had a higher survival rate than HIV-infected CM patients (Liu et al., 2017). Higher proportion of HIV-uninfected CM patient might contribute higher 90-day survival rate.

There were some limitations in our study. First, our study was a retrospective study, although we tried to reduce certain biases by using a PSM method. Therefore, a prospective study is needed to further address the role of AmB in treatment with AmB + 5FC ± Flu. Second, our data were based on a small sample size and a short follow-up duration. Consequently, a study based on a larger sample size and prolonged observation is needed in the future. Third, the influence of 5FC and Flu on the outcomes of patients was not discussed. Nevertheless, we and other researchers understand the potential effects of 5FC and Flu upon patient outcomes. For example, a study indicated that the addition of 5FC and a short course of AmB therapy to high-dose Flu therapy significantly enhanced early fungicidal activity and may be associated with favorable trends in survival (Jackson et al., 2012).

In summary, our study suggested that compared with induction treatment with AmB + 5FC, the addition of Flu to AmB + 5FC for induction treatment did not improve the clinical outcomes of Chinese CM patients. The short-term (less than 14 days) together with low dose (less than 0.7 mg/k/d) usage of AmB is associated with a higher mortality risk in patients. The use of low dosages of AmB (such as 0.5–0.7 mg/kg/d) and a prolonged course (more than 14 days) in an AmB + 5FC ± Flu regimen were safer and of comparable efficacy in Chinese CM patients.

## Data Availability

All datasets generated for this study are included in the manuscript and/or the supplementary files.

## Ethics Statement

The study protocol was in accordance with the Declaration of Helsinki 1975 guidelines and was approved by the Ethics Committee of the First Affiliated Hospital, College of Medicine, Zhejiang University (Hangzhou, China) (No. 2017-688) and all data analyzed were anonymous.

## Author Contributions

LX, JH, and BZ conceived and designed the study. RT, JW, XD, and CH collected the data. LX analyzed the data. YH followed up the study. LX and YC wrote the manuscript.

## Conflict of Interest Statement

The authors declare that the research was conducted in the absence of any commercial or financial relationships that could be construed as a potential conflict of interest.
